# Migraine and neuroinflammation: the inflammasome perspective

**DOI:** 10.1186/s10194-021-01271-1

**Published:** 2021-06-10

**Authors:** Oguzhan Kursun, Muge Yemisci, Arn M. J. M. van den Maagdenberg, Hulya Karatas

**Affiliations:** 1 Department of Neurology, City Hospital, Ankara, Turkey; 2 Institute of Neurological Sciences and Psychiatry, Hacettepe University, Ankara, Turkey; 3 Department of Neurology, Faculty of Medicine, Hacettepe University, Ankara, Turkey; 4 Department of Human Genetics, Leiden University Medical Centre, Leiden, The Netherlands; 5 Department of Neurology, Leiden University Medical Centre, Leiden, The Netherlands

**Keywords:** Migraine, Cortical spreading depolarization, Neuroinflammation, Inflammasome, Mitochondrial DNA, Comorbidity

## Abstract

**Background:**

Neuroinflammation has an important role in the pathophysiology of migraine, which is a complex neuro-glio-vascular disorder. The main aim of this review is to highlight findings of cortical spreading depolarization (CSD)-induced neuroinflammatory signaling in brain parenchyma from the inflammasome perspective. In addition, we discuss the limited data of the contribution of inflammasomes to other aspects of migraine pathophysiology, foremost the activation of the trigeminovascular system and thereby the generation of migraine pain.

**Main body:**

Inflammasomes are signaling multiprotein complexes and key components of the innate immune system. Their activation causes the production of inflammatory cytokines that can stimulate trigeminal neurons and are thus relevant to the generation of migraine pain. The contribution of inflammasome activation to pain signaling has attracted considerable attention in recent years. Nucleotide-binding domain (NOD)-like receptor family pyrin domain containing 3 (NLRP3) is the best characterized inflammasome and there is emerging evidence of its role in a variety of inflammatory pain conditions, including migraine. In this review, we discuss, from an inflammasome point of view, cortical spreading depolarization (CSD)-induced neuroinflammatory signaling in brain parenchyma, the connection with genetic factors that make the brain vulnerable to CSD, and the relation of the inflammasome with diseases that are co-morbid with migraine, including stroke, epilepsy, and the possible links with COVID-19 infection.

**Conclusion:**

Neuroinflammatory pathways, specifically those involving inflammasome proteins, seem promising candidates as treatment targets, and perhaps even biomarkers, in migraine.

## Introduction

Migraine pathophysiology is the result of interactions of neurons, glial cells, the vasculature, and inflammatory signaling, cumulating into a highly debilitating neurological disorder that is characterized by recurrent unilateral headaches that last 4 to 72 h and that are accompanied by other features, such as nausea and photo/phonophobia [1]. Two main types of migraine are distinguished based on the presence of an aura (preceding the headache) and that is characterized by slowly spreading visual and sensory disturbances, hence [1], migraine with aura (MA) and migraine without aura (MO). Migraine affects over 15% of the general population and is among the most prevalent and disabling chronic disease conditions, also in terms of morbidity and lost productivity due to absenteeism of work [2]. Migraine occurs three times more often in women than men [3]. The high economic burden of migraine loads heavily on healthcare systems of countries worldwide [4]. Therefore, reducing the burden of migraine remains key but the development of better treatment options is hampered by our incomplete understanding of the pathophysiology of migraine through basic and clinical research models.

Neuroinflammation is regarded as the adaptive reaction initiated by noxious stimuli, such as infection, injury and tissue stress, and plays an important role in the pathophysiology of various diseases of the central nervous system [5]. In the context of migraine, neurogenic neuroinflammation is defined by inflammatory reactions in the central and peripheral parts of the trigeminovascular system in response to neuronal activity [6]. Although the term is mainly applied to central nervous system (CNS) components it is also relevant to peripheral nervous system (PNS) structures, such as the trigeminal ganglion. The immune system is divided into a more primitive innate immune system and an acquired or adaptive immune system, each of which contain both humoral and cellular immune elements. Over the past decade, there has been a surge in information regarding the composition and actions of the innate immune system of the CNS, which includes microglia, trafficking macrophages and astrocytes [7]. The interactions between innate immune cells and infiltrating adaptive immune cells (T and B lymphocytes) in the CNS have become better understood in recent years, and this has prompted the recognition that each of these cell types contributes to the development of inflammation in the CNS — a highly orchestrated response by the immune system to infectious or non-infectious (sterile) stimuli, which are involved in various diseases including MA, stroke, and multiple sclerosis [7]. Inflammation in the CNS has both pathogenic and protective effects depending on the biological circumstances, tissue homeostasis, involved cell types and inflammatory elements.

In this review, we will discuss, from the inflammasome perspective, the link between migraine and neuroinflammatory signaling in the brain parenchyma, how genetic factors make the brain vulnerable to CSD, and the relation with diseases comorbid with migraine, foremost stroke, and epilepsy as well as possible links with COVID-19 infection.

## Pathophysiology of migraine

The pathophysiology of migraine has yet to be fully elucidated, but it is believed that the headache is caused by activation of the trigeminovascular system [8, 9], which consists of peripheral trigeminal nerve endings innervating the dural vasculature. The trigeminal nerve mediates pain sensation from cranial structures in the anterior two-third of the head and plays a crucial role for the headache phase. Upon nerve activation, trigeminal nociceptors mediate release of compounds, such as calcitonin gene-related peptide (CGRP), substance P (SP) and nitric oxide into the perivascular space [10]. Stimulation of trigeminal neurons in ganglia of experimental animals results in a sterile neurogenic inflammation that is characterized by release of CGRP and SP, vasodilation, increase in blood flow, and protein extravasation in the dura mater [11]. Activation of meningeal perivascular trigeminal nociceptors by various compounds, including potassium and arachidonic acid (AA), can initiate signal transmission orthodromically to pain processing structures, while inducing antidromic neurogenic inflammation in the dura mater [10]. Signals from perivascular neurons result in a downstream cascade of events causing the release of vasoactive inflammatory mediators, inflammation in the meninges, and sensitization of pain-related brainstem areas [12]. As such, the headache phase of a migraine attack is thought to result from the activation of nociceptors that transmits signals to trigeminal bipolar neurons. The signals are then further relayed to thalamic and cortical areas to produce the sensation of pain [10, 11].

The immune system plays a key role in migraine pathogenesis. Various cytokines, including tumour necrosis factor (TNF), interleukin 1 (IL-1) and adiponectin, have been implicated in inflammation, modulation of the pain threshold, trigeminal nerve fiber sensitization, and ultimately the precipitation of migraine [13]. Headache can be induced after TNF injection, whereas TNF antibody was shown to reduce pain in humans [13]. Plasma levels of both pro- and anti-inflammatory cytokines are increased during migraine attacks. TNF levels increase after migraine pain onset and decrease progressively over time after the onset of the attack [14]. Aydin et al. showed that the levels of TNF-α, IL-6 and IL-10 change in the blood of patients with migraine [15]. TNF-α and IL-6 levels were significantly higher in migraine patients compared to healthy controls during and between attacks. During attacks, IL-10 levels were higher than outside an attack and than in healthy controls. Yilmaz et al. reported on the genotypic distribution of the TNF-α-308 G/A and IL-1β + 3953 C/T polymorphisms in migraineurs compared to controls and demonstrated that the frequency of the TNF-α-308 GG genotype was higher in the control group than in the MO group [16]. The TNF-α-308 G allele was overrepresented in the control group, whereas the TNF-α-308 A allele was more prevalent in the MO group. In addition, there was a significant increase of the IL-1β + 3953 T allele among MO cases compared with controls [16]. These studies suggests that there is a possible contribution of peripheral cytokines, including TNF-α, IL-6, IL-10 and IL-1β, to migraine headache pathogenesis.

### Cortical spreading depolarization

Whereas it is less well understood how trigeminal activation triggers the migraine headache, there is accumulating evidence that cortical spreading depolarization (CSD) is the electrophysiological correlate of the migraine aura [17, 18]. CSD is a slowly propagating wave of neuronal and glial depolarization accompanied by massive ion fluxes, spreading through the cortex, and that is followed by a long-lasting suppression of neuronal activity [17, 19–21]. It has been suggested that CSD also plays a critical role in headache initiation via parenchymal neuroinflammatory signaling [22, 23], at least in patients with MA. CSD is associated with opening of neuronal Pannexin1 mega channels causing caspase-1 activation with concomitant IL1-β and high mobility group box 1 (HMGB1) release, which initiates parenchymal inflammatory pathways via nuclear factor-kappa B (NF-κB) translocation to the nucleus of astrocytes, with subsequent expression of inflammatory genes, such as cycylooxygenase and inducible nitric oxide synthase, in astrocytic processes that form the glia limitans, and the release of potassium and AA into the perivascular space [10, 23]. This cascade of events may provide the stimulus for sustained trigeminal nerve activation around meningeal vessels [10, 23]. Various studies using different CSD induction methods that include topical potassium chloride (KCl) application to the cortex, pinprick, electrical stimulation, and optogenetics have shown that CSD can induce neuroinflammatory responses in the rodent brain [24–28]. CSD was also shown to transiently activate matrix metalloproteinase-9 activation leading to a temporarily breakdown of the blood-brain barrier (BBB) [21], thus providing a possible explanation how intracellular nociceptive and vasoactive molecules may induce neuroinflammation and find a way to pass through the BBB and reach perivascular trigeminal nociceptors in the dura mater [23, 24, 29], although it is debated to what extent BBB breakdown occurs in migraine patients [30].

A clear link between genetic susceptibility and neuroinflammation comes from studies in familial hemiplegic migraine (FHM), a monogenic subtype of MA [1]. A FHM1 knock-in mouse model that expresses the R192Q missense mutation in the α_1A_ subunit of voltage-gated Ca_V_2.1 Ca^2+^ channels resulting in a gain of channel function with increased neurotransmission [31, 32] and an increased susceptibility to experimental CSD [31–33]. The link with neuroinflammation is most evident by the observation that naïve mutant mice exhibit signs of brain reactive astrogliosis and microglia activation [34]. In addition, the mutant mice showed basal macrophage activation and intensified neuronal currents mediated by purinergic P2X3 receptors in trigeminal ganglion cultures [35]. Another study in the same mutant mice demonstrated that there is a constitutive up-regulation of P2X3 receptors in trigeminal ganglion cultures, which makes these neurons more responsive to extracellular ATP [36]. In support of the inflammatory nature of the FHM1 knock-in brain, trigeminal ganglion neurons of R192Q mice showed a higher expression of P2X7 receptors and enhanced responses to benzoyl-ATP than wild-type mice, which was prevented by prior administration of a Ca_V_2.1 blocker, small interfering RNA (siRNA)-based silencing of P2X7 receptors, or a P2X7 antagonist [37]. Mutant trigeminal ganglion neurons also revealed higher basal expression of bradykinin, an algogenic mediator that potentiates purinergic P2Y receptors, and stimulated CGRP release [38]. Besides, it was demonstrated that TNFα expression and macrophage presence were significantly higher in R192Q mutant trigeminal ganglia ganglia after an inflammatory reaction was induced by lipopolysaccharide injection [39]. Together, the studies support the notion that neuroinflammation is relevant for the mutant migraine mouse brain.

Proof of a direct link between CSD-induced neuroinflammation and migraine in humans came from a recent PET/MRI study [40]. In that study, brain scans with ^11^C-PBR28, a marker of inflammatory glial activation, were obtained from MA patients and healthy controls. Migraineurs revealed a surprising increased widespread bilateral neuroimmune activation in various brain areas, including thalamus and primary and secondary somatosensory and insular cortices, which are involved in pain processing [40]. The neuroimmune activation was thought to be specific to migraine, and not pain in general, since patients suffering from chronic lumbar pain did not reveal tracer uptake in pain processing areas [41]. Moreover, the tracer signal intensity was positively associated with the frequency of MA attacks [40, 41], suggesting that repeated CSDs in MA may induce widespread neuroinflammatory activity.

## Inflammasomes in migraine

The link between migraine and the inflammasome is starting to draw attention, also in light of the occurrence of migraine-like attacks seen with various autoinflammatory diseases, foremost cryopyrin-associated periodic fever syndromes (CAPS), a broad spectrum of rare autoinflammatory diseases. CAPS is an autosomal dominant autoinflammatory disorder associated with gain-of-function mutations in the nucleotide-binding domain (NOD)-like receptor family pyrin domain containing 3 (*NLRP3)* gene, which eventually cause an extreme production of IL-1β and systemic inflammation [42, 43]. Typical systemic features include fever, urticarial rash and arthralgia, and, finally, amyloidosis. There are also multiple neurological presentations, such as migraine-like headache, sensorineural hearing loss, aseptic meningitis, myalgia and optic nerve involvement [42, 44]. Interestingly, a prospective, open-label, long-term study in 43 patients with severe CAPS demonstrated that treatment with anakinra (an IL-1 receptor antagonist) significantly decreased CNS inflammation and headache in pediatric patients [45].

The innate immune system is a cellular defense of organisms confronted with sterile and infectious insults and responds in a rapid and coordinated manner. The detection of pathogenic signals is recognized by pattern recognition receptors (PRRs) that sense pathogen-associated molecular patterns (PAMPs) and host- or environment-originated danger-associated molecular patterns (DAMPs). In the CNS, these PRRs are primarily expressed in microglia, astrocytes, and macrophages, although also oligodendrocytes, neurons, and endothelial cells express PRRs [7, 46]. PRRs recognize danger signals, such as sterile tissue damage, metabolic alterations, and general stress in tissues and start an immune reaction to maintain tissue homeostasis. PRRs are located at the plasma membrane and in the cytoplasm. Membrane-bound PRRs include Toll-like receptors (TLRs), which sense extracellular signals. Instead, nucleotide-binding domain and leucine-rich repeat-containing receptors (NLRs) and a melanoma 2 (AIM2)-like receptors (ALRs) are PRRs that are present in the cytoplasm and sense intracellular signals. The intracellular PRRs form “inflammasomes” as part of the innate immune response.

### General concepts of inflammasomes

**Fig. 1:**
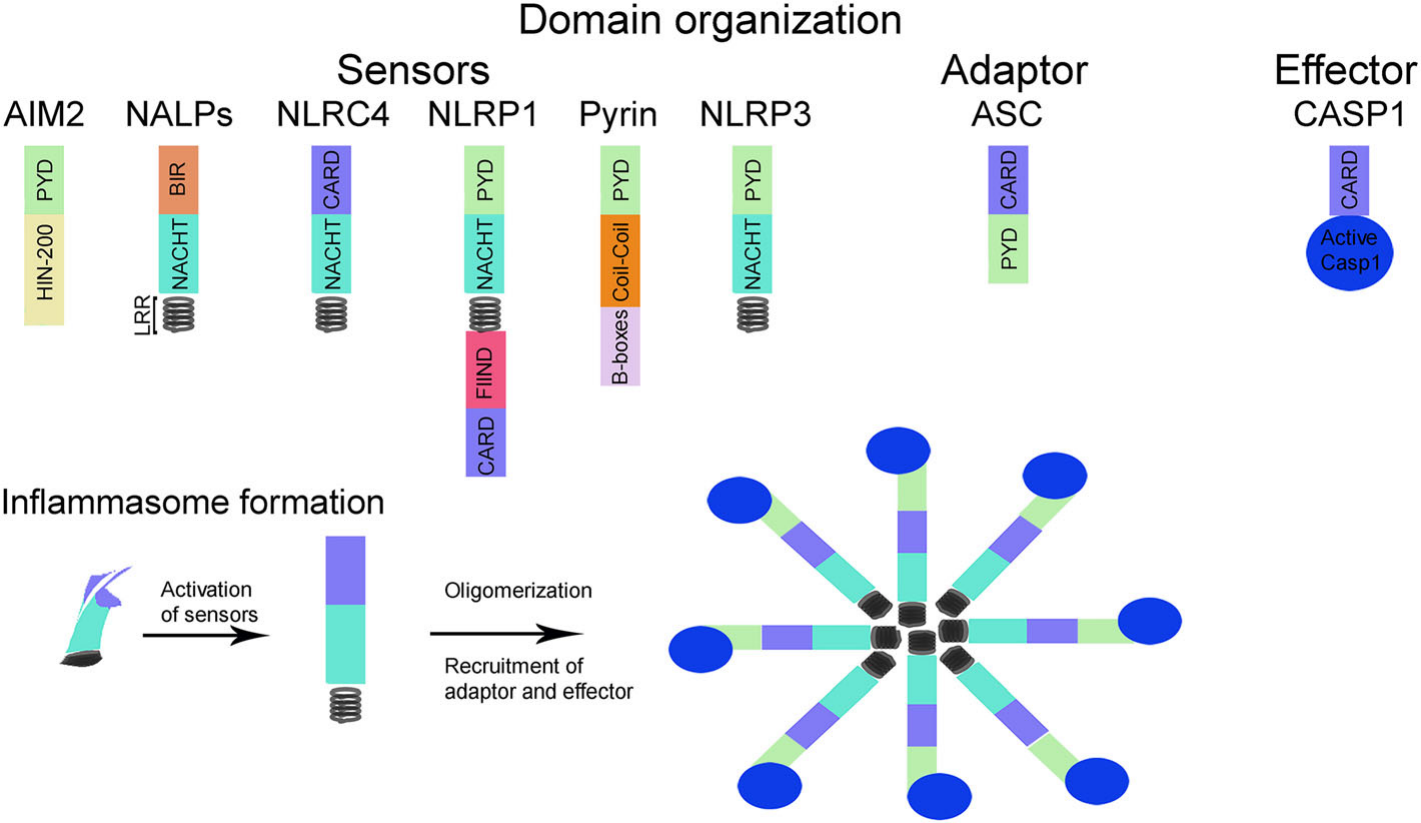
Simplified scheme of inflammasome elements, types and formation. Domain organization and basic inflammasome formation. At the molecular level, assembly of inflammasome signaling complexes is mediated through interactions between homotypic protein domains. The domains of AIM2, NALPs, NLRC4, NLRP1, Pyrin, NLRP3, ASC, and CASP1 are shown here. The inflammasome is composed of a sensor, adaptor, and effector protein. Several inflammasomes contain a PYD, responsible for the recruitment of the adaptor protein ASC. After activation, inflammasomes using this adaptor interact with ASC through PYD–PYD interactions, forming large, filamentous oligomers. ASC then recruits CASP1 across CARD–CARD interactions. NLRP1 and NLRC4 may directly interact with CASP1 by CARD–CARD interactions, though these NLRs could also interact with ASC via CARD–CARD interactions. After activation, the sensor oligomerizes and recruits the adaptor and effector proteins to the inflammasome complex. ASC, apoptosis-associated speck-like protein containing a caspase recruitment domain; AIM2, absent in melanoma 2; BIR, baculoviral inhibitor of apoptosis protein repeat; FIIND, function-to-find domain; CARD, caspase recruitment domain; CASP, caspase; LRR, leucine-rich repeat; NALP, Nucleotide-binding oligomerization domain, Leucine-rich Repeat and Pyrin domain containing; NLRC4, NLR family CARD domain-containing protein 4; NLRP1, nucleotide-binding domain (NOD)-like receptor family pyrin domain containing 1; NLRP3, nucleotide-binding domain (NOD)-like receptor family pyrin domain containing 3; PYD, pyrin domain

Inflammasomes are cytosolic multiprotein complexes that, when assembled, lead to cleavage of pro-inflammatory caspase-1. Subsequently, cleaved active caspase-1 causes proinflammatory cytokines (IL-1β and IL-18) maturation and secretion from the cell [47, 48]. Inflammasomes are classified according to their signal initiating receptor [49]. Main inflammasome components are the cytosolic NLR, AIM-like receptor (ALR), and pyrin receptors, the adaptor apoptosis-associated speck-like protein containing a caspase recruitment domain (ASC), and pro-caspase-1 (Fig. 1). The ASC is composed of a pyrin domain (PYD) and a caspase recruitment domain (CARD) and functions as an adaptor complex that associates the pyrin domain of NLR or pyrin with the CARD of pro-caspase-1 (Fig. 1). Each inflammasome is named after its NLR or ALR protein scaffold [49]. NLRC4 and NLRP1b inflammasomes may directly bind pro-caspase-1 via CARDs without an ASC domain [50]. Inflammasomes assembly and activation requires a specific stimulus, such as bacterial toxins, intracellular flagellin, bacterial pathogens, or cytosolic double-stranded DNA [51]. The most studied inflammasome, NLRP3, reacts to many signals that include bacterial, fungal, and viral PAMPs, DAMPs, such as ATP and uric acid crystals, and crystalline as well as aggregated substances, such as asbestos, silica, α-synuclein and amyloid-β fibrils, and intracellular homeostatic changes, such as increased potassium efflux, mitochondrial dysfunction, lysosomal damage, and ROS production [49, 51, 52]. A two-step process is necessary for NLRP3 activation, which is unique among inflammasomes. A priming signal causes NF-κB-dependent transcriptional upregulation of NLRP3 and pro-IL-1β [53]. Then, one of the activation signals mentioned above induces oligomerization and activation of the NLRP3 inflammasome complex. In contrast to NLRP3, other inflammasome complexes do not need a priming signal to induce inflammasome activation and cytokine release.

Inflammasome complex elements exist in all cells, but how and which of them assemble together depends on the type of stimulus and the homeostasis of the cell. Therefore, multiple types of inflammasomes may be present in one cell type. For example, whereas it was first suggested that NLRP3 inflammasomes are exclusive to microglia, recent studies demonstrated that NLRP3 inflammasome complex may also form in other brain cells, including oligodendrocytes, astrocytes and neurons [52, 54, 55]. This notion may not only change our view on how inflammasomes play a role in neurological disorders, but also likely affects the design of inflammasome-related therapeutic target studies.

**Fig. 2:**
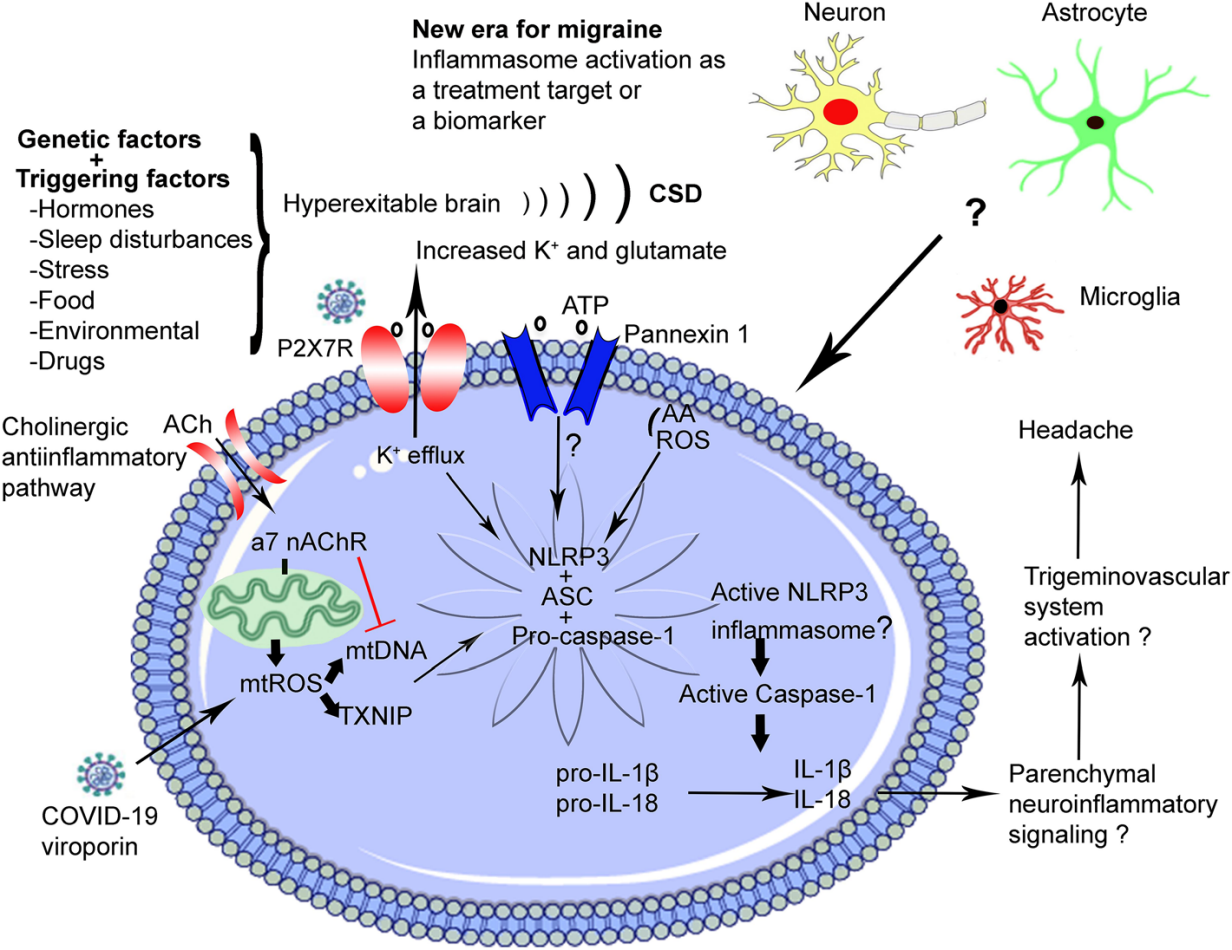
New era for migraine: Inflammasome activation as a treatment target or a biomarker. In the background of genetically hyperexcitable brain, triggering factors induce CSD which results in Pannexin-1 megachannel opening and K^+^ efflux from P2X7R, consequently creates stress on the cell. Reactive oxygen species (ROS) from membranous Arachidonic acid (AA) and mitochondria respond to this cellular stress via mtROS production which is sensed by mtDNA and TXNIP. K^+^ efflux, mtDNA and Pannexin-1 megachannel opening cause the assembly of inflammasome complex elements (NLRP3, ASC, pro-caspase-1) and activation of caspase-1 which cleaves pro forms of IL-1β and IL-18 and ignites parenchymal neuroinflammatory signaling to alert adjacent cells. This cascade eventually reaches the trigeminovascular system around meningeal vessels which results in activation of this system and consequently headache. COVID-19 viroporins induce NLRP3 inflammasome formation via increase in mitochondrial ROS production. This may be an overlapping mechanism in migraine and COVID-19 headache. On the contrary, ACh, main actor in cholinergic anti-inflammatory pathway, inhibits NLRP3 inflammasome by decreasing mtDNA release through binding to mitochondrial α7 nAChR

Clinical and experimental studies investigating neuroinflammation in migraine demonstrated the role of inflammasome only indirectly, that is via inflammasome complex players including IL-1β, IL-18, and caspase-1 (Fig. 2). For example, it was shown that IL-1β is increased in internal jugular blood during a migraine attack as well as between attacks [56]. Furthermore, serum concentrations of IL-1β, IL-6, IL-8, and TNF-α were found increased during migraine attacks [57]. IL-1β-induced pro-inflammatory process mediates the activation of trigeminal satellite cells and stimulates cross excitation of satellite glial cells and neurons in the trigeminal ganglion (TG) [58]. Another study demonstrated higher levels of IL-1 receptor antagonist (IL-1RN), transforming growth factor β1, and monocyte chemoattractant protein 1 in cerebrospinal fluid of migraine patients compared to controls [59].

Until now, few rodent studies have investigated the link of the inflammasome and migraine. One study showed the involvement of the NLRP3 inflammasome pathway in the peripheral TG response of the rat inflammatory dural stimulation-induced model of intracranial pain [60]. Another study demonstrated that the expression of the NLRP3 inflammasome was upregulated in a migraine-relevant pain mouse model (that is pain was induced by recurrent NTG stimulation [61]), shown by an increase in NLRP3 expression that was associated with IL-1β activation. In that study, blockade of NLRP3 and IL-1β both improved hyperalgesia and inhibited the increase of biomarkers related to central sensitization of chronic migraine, such as p-ERK, c-Fos and CGRP in the trigeminal nucleus caudalis.

A recent review suggested that D-β-hydroxybutyrate (D-BHB), one of ketone bodies, is implicated in migraine pathophysiology, that is in mitochondrial function, oxidative stress, cerebral excitability, inflammation, and the gut microbiome [62]. It was shown that D-BHB prevented the decline in intracellular K^+^ levels and prevented activation of the NLRP3 inflammasome [63]. NLRP1 (NALP1) has been associated with different pain syndromes, including neuropathic pain, complex regional pain syndrome, adjuvant arthritis, chronic pelvic pain syndrome and may serve as a new target for pain therapy [64]. A recent study showed that activation of the NLRP2 inflammasome leads to activation of dorsal root ganglia neurons and the development of pain hypersensitivity [65]. However, there is no data implying a relation between NLRP1 or NLRP2 inflammasome and migraine and/or CSD. Hence the role of NLRP1, NLRP2, NLRP3, AIM2 and other inflammasomes in MA-associated parenchymal neuroinflammation, for instance in response to CSD, needs to be investigated in future studies.

## Migraine and neuroinflammation-related genes

Migraine is a complex genetic disease, which means that it is brought about by a combination of multiple genetic and environmental factors [66, 67]. Family and epidemiological studies have revealed an increased risk for family members for the common forms of migraine with heritability estimates of 34–64% [66–68]. More specifically, a first-degree relative of a MO proband is twice as likely to also suffer from MO (and 1.4 times as likely to have MA [69]), whereas a first-degree relative of a MA proband is 4 times more likely to have MA (but does not have an increased risk of MO) [70, 71]. In addition to common polygenic migraine, various types of monogenic migraine - indicating that the presence of a single genetic factor suffices to bring about disease in a patient -, of which FHM is the most studied [1, 9]. Until now, three undisputed hemiplegic migraine genes, *CACNA1A* (FHM1), *ATP1A2* (FHM2), *SCN1A* (FHM3), have been identified; despite recent technical advances in whole genome/whole exon next generation sequencing no additional hemiplegic migraine genes have been identified [69]. A likely explanation is that there is considerable heterogeneity among patients with hemiplegic migraine and it was shown that chances of finding a causal mutation are higher in those with a lower age of disease onset, especially when multiple family members are affected and when attacks are more severe (e.g. mild head trauma-triggered, confusion, extensive motor weakness, cerebellar ataxia) [72].

### Evidence from studies in experimental animals

Several studies investigated the relation between CSD and neuroinflammation, mainly by investigating changes in gene expression of inflammatory genes after CSD [22, 73, 74]. For instance, *TNFα* and *IL-1β* mRNA levels were transiently increased after KCl-induced CSD in the rat ipsilateral cortex [73]. A recent study showed that multiple CSDs, induced by optogenetics in Thy1-channelrhodopsin-2 transgenic mice non-invasively through intact skull, caused increase in cortical *IL-1β, chemokine (C-C motif) ligand 2*, and *TNFα* mRNA expression peaking at 1, 2 and 4 h, respectively. This response was decreased in IL-1 receptor 1 knockout mice, implicating IL-1β as an upstream mediator [25].

A re-analysis of published data from Eising et al. [75], in which gene expression changes 24 h after KCl-induced CSD were investigated in the transgenic knock-in mouse model, in which the human FHM1 R192Q missense mutation was introduced [32], showed increased expression of IL-1 receptor antagonist (IL-1RN), and that expression of IL-6 was higher in the brains of FHM1 mutant mice compared to wild-type mice, whereas IL-2, IL-4, IL-10 and IL-13 did not reveal differential genotypic expression [76]. In that study it was speculated that IL-1RN and IL-6 may exert a homeostatic role aimed at counteracting ongoing immunoinflammatory events. In the original paper by Eising et al. [75], it was reported that CSD events produce a delayed unique inflammatory signature typified by interferon-mediated inflammatory signaling, as evidenced by an overrepresentation of interferon-related transcription factor binding sites (IRF, ISRE and ICSBP) in the promoter regions of identified genes. In addition, CSD caused a pronounced sustained up-regulation of genes, including *Cd53*, *Ms4a6d*, *Anxa2*, *Ccl2*, *Vim*, *C3ar1* and *Timp1*, that are key drivers of inflammatory signaling [77]. The combined results indicate a key role for a neuroinflammatory mechanism in response to CSD that is specific for FHM1 mutant mice.

### Evidence from genetic studies in humans

Human genetic studies also revealed an association of migraine and inflammation. For instance, a recent genome-wide association analysis (GWAS) that compared genetic data of 59,674 migraine cases and 316,078 control individuals yielded 38 genomic susceptibility loci in human, among which 5 genes, i.e. *TSPAN2*, *MEF2D*, *NLRP1*, *JAM3* and *NOTCH4*, that are associated with inflammation [78]. The gene product of *TSPAN2* has a role in microglial cells and astrocytes to maintain healthy CNS and regulate neuroinflammation, among other functions [79]. The *MEF2D* gene encoded protein functions to protect microglial cells from inflammation-induced toxicity [80]. The *NLRP1* gene codes for a well-known sensor component of the NLRP1 inflammasome and has an essential role in innate immunity and inflammation [7]. Of note, activation of the NLRP1 inflammasome is necessary for HMGB1 secretion, which together with active cytokines, induces inflammatory signaling [81]. The product of the *JAM3* gene is important for cell-cell adhesion at endothelial tight junctions as well as the regulation of leukocyte recruitment to sites of inflammation [82]. And finally, the *NOTCH4* gene product regulates the inflammation response [83]. Further genetic evidence for involvement of neuroinflammation in migraine comes from a comparison of genetic data of 4505 MA and 4038 MO (and respective control sets) in a GWAS setting that showed that both migraine types are more alike than different and that among the genetic overlap of both migraine types there was an enrichment of genes related to inflammation, in addition to genes that play a role in cardiovascular system and connective tissue [84].

## Mitochondria, migraine and the inflammasome

Another avenue of exploration of migraine susceptibility is through the genetic analysis of mitochondrial DNA (mtDNA), which has been linked to various complex genetic traits [85]. Abnormal mitochondrial function can result in high intracellular Ca^2+^ levels, excessive production of free radicals, and deficient oxidative phosphorylation, which ultimately causes energy failure in neurons and astrocytes, which have been hypothesized to play a role in migraine pathophysiology [86–88]. For instance, mitochondrial abnormalities have been found in migraine patients, as evidenced by direct observations in muscle biopsies that revealed ragged red and cytochrome-c oxidase-negative fibers, the accumulation of subsarcolemmal mitochondria, and the demonstration of giant mitochondria with para-crystalline inclusions [89, 90]. In addition, there have been genetic studies that linked DNA variants in mitochondria to migraine. Common mtDNA polymorphisms (16519C-T and 3010G-A) have been associated with pediatric cyclic vomiting syndrome and migraine [91]. In addition, various *POLG* missense mutations have been associated with migraine [92]. Still, the importance of these variants for migraine pathophysiology is not undisputed. Perhaps not unexpectedly, a recent mitochondrial GWAS of migraine was not successful in identifying a genetic factor [93].

Regardless, there is little doubt that mitochondria, already given their major role in supplying energy to the brain, must play an important role in migraine pathophysiology. In this respect it is relevant that the prevalence of migraine in a clinical study including one progressive external ophthalmoplegia, twelve myoclonic epilepsy with ragged red fibers, eight mitochondrial encephalomyopathy, lactic acidosis and stroke-like episodes [MELAS], two mitochondrial neurogastrointestinal encephalomyopathies, and 13 other mitochondrial diseases was roughly twice that of migraine in the general population [94]. For example, there was a high prevalence of migraine headache in MELAS patients carrying the m.3243A > G mutation [95]. This mutation in vascular endothelial and smooth muscle cells, neurons and glial cells induces long-term exposure of toxic substances, such as reactive oxygen species (ROS), which may contribute to the occurrence of migraine at a later age. In addition to the increased production of ROS, narrowing the vascular lumen and the consequent hypoxia/ischemia, lipid peroxidation, altered glutamate metabolism and ionic homeostasis may be relevant as trigger of CSD. These pathways, including the inflammation pathways stimulated by ROS induction, have been proposed as new targets for innovative classes of drugs to treat mitochondrial migraine in m.3243A > G patients [95].

Mitochondria also interact with the immune system, more precisely with inflammasome induction. This is because mitochondria can generate mitochondrial ROS (mtROS), which damages mtDNA and interacts with the NLRP3 inflammasome during inflammatory reactions [96, 97]. This mtROS overproduction is sensed by thioredoxin-interacting protein (TXNIP) or mtDNA, which then binds to the leucine-rich repeat region of NLRP3 and thus leads to NLRP3 inflammasome activation [97]. The formation of ROS and the release of mtDNA from mitochondria to the cytoplasm are among the cellular signals that play an important role in NLRP3 inflammasome activation [47]. Besides this, it was shown that ROS generation and inflammasome activation is decreased by rotenone, a mitochondrial complex I inhibitor [98]. Moreover, a specific mitochondria ROS scavenger, mito-TEMPO, abolished mtROS release, thereby inhibiting NLRP3 inflammasome activation and reducing the upregulation of IL-1β and IL-18 induced by ethanol or lipopolysaccharide/ATP [99]. Of note, the destructive effects of NLRP3 inflammation, which is activated in pathological processes, are associated with oxidative stress and mitochondrial dysfunction [100, 101]. Therefore, mitochondrial stress-induced mtDNA-triggered NLRP3 inflammasome activation may be an additional mechanism leading to neuroinflammatory signaling pathway induced by CSD.

## Cholinergic anti-inflammatory pathway and the inflammasome

Cholinergic anti-inflammatory pathway is a regulatory pathway that requires the vagus nerve produce acetylcholine (ACh), and α7 nicotinic acetylcholine receptor (α7 nAChR) expressed in cytokine-producing immune cells. Stimulation of the vagus nerve increases ACh levels, which prevents excessive proinflammatory cytokine production, and the mechanism of how ACh inhibits inflammatory reaction was demonstrated by Lu et al. [102]. Extracellular ATP results in rapid influx of ACh into cytoplasm where ACh prevents mitochondrial DNA release, which is an NLRP3 ligand through α7 nAChR, and subsequently inhibits NLRP3 inflammasome activation in LPS-primed mouse peritoneal macrophages [102]. In that setup cholinergic receptor agonists or vagus nerve stimulation (VNS) significantly inhibit inflammasome activation, whereas genetic ablation of α7 nAChR significantly enhances inflammasome activation. FDA approved VNS for the treatment of migraine pain in adults in 2018. There are multiple possible mechanisms of VNS related to migraine. Recently, it was shown that VNS inhibits CSD and that this effect is due to the activation of afferent fibers in the cervical vagus nerve, and that it involves nucleus tractus solitarius and probably noradrenergic and serotonergic signalling [103]. Other possible mechanisms include inhibition of mechanical nociception, repressed expression of proteins associated with peripheral and central trigeminal sensitization, and decreased trigeminal nociception by suppressing the rise in glutamate after nitric oxide treatment [104, 105]. VNS shows that cholinergic anti-inflammatory pathway is important in migraine pathophysiology via reduction of CSD susceptibility, inhibition of mtDNA release, and the NLRP3 inflammasome (Fig. 2).

## Comorbidity of migraine with other diseases

Various medical conditions are more common in people with migraine compared to the general population [106]. According to the “Migraine in America Symptoms and Treatment” (MAST) study, headache pain intensity and monthly headache days are associated with an increased risk for many diseases, among which depression, anxiety, stroke and epilepsy [107]. Several theories have been proposed to explain the etiology behind the association with the comorbid conditions, including shared environmental and/or genetic risk factors [108]. Despite some promising results from comparing GWAS data of migraine and comorbid diseases, as was performed within *the Brainstorm consortium* [109], we are still only at the beginning of unraveling the shared genetic underpinning of brain diseases. Clearly, it is important to understand better the comorbidities of migraine and recognize the genetic contribution, already because it may lead to better treatment strategies for patients.

Parenchymal neuroinflammation seems a disease pathway shared between migraine and comorbid diseases, such as stroke, epilepsy, and possibly COVID-19 infection. Both experimental and clinical studies have pointed at a clear link between migraine and stroke [110]. Both preclinical models and clinical data provide possible explanations for the association between the two most common neurological disorders, migraine and stroke [110, 111]. The increased risk of ischemic stroke in migraineurs is elucidated by shared genetic factors, cardiovascular dysfunction, coagulopathies, excitotoxicity amongst increased inflammation [110]. Migraine-related ischemic complications have been contributed to neuroinflammation, which seems to suggest common underlying disease mechanisms. The interplay between peripheral and central inflammasome-mediated pathways are intermingled in pathophysiological mechanisms in the brain, not only in the context of migraine but also stroke. That is, the roles of NRLP1, NRLP3, NRLP4 and AIM2 inflammasomes in stroke, and its outcome, were determined via various in vitro and in vivo settings [112–117]. The sudden cessation of blood in stroke leads to a decrease in ATP that activates AMP-activated protein kinase (AMPK) and subsequently the activation of NLRP1 [118, 119]. Actually, a physiological ATP concentration is anticipated to inhibit NLRP1, and activation of AMPK is not sufficient to activate NLRP1 inflammasome, and the decrease in ATP concentration is essential [118]. In ischemic stroke, the increase in cytosolic Ca^2+^ and the decrease in K^+^ is determined to activate NLRP3. Aberrant Na^+^/K^+^-ATPase pump function, via low ATP levels, can cause differences in the concentrations of various ions, causing the activation of NLRP1 and NLRP3 inflammasome assembly [120, 121]. This suggests that a reduction in intracellular ATP is sensed by NLRP1 and NLRP3 [118, 119]. Recently, in the transient middle cerebral artery occlusion model, gene and protein levels of inflammasomes were determined in mice soon after the start of ischemia and recanalization, which represent the hyperacute and acute phases in ischemic stroke patients [117]. It was shown that in early stroke NLRP1, NLRC4 or AIM2 did not have significant effects, while NLRP3 was predominantly expressed in ischemic neurons, and its inhibition improved neurologic outcome with a reduction in infarct size [117]. Although the impact of the other inflammasomes should also be taken into account, early neuronal NLRP3 up-regulation seemed the driver of leukocyte infiltration, and BBB disruption. However, it should be kept in mind that immune and glial cells also express inflammasomes and may contribute to the post-ischemia inflammation after early neuronal alarm [44, 122, 123].

Migraine and epilepsy share clinical features, are caused - to certain extent - by similar underlying pathophysiological mechanisms, and antiepileptics are effective in treating patients with both disorders [124, 125]. Investigations in experimental epilepsy models may further the understanding of the close interplay between increased neuronal excitation and inflammation [126]. The neuropathological examination of hippocampal sections from patients with refractory mesial temporal lobe epilepsy (mTLE) revealed severe neuronal loss and increased levels of NLRP1 and caspase-1 compared to controls [127]. Also in an experimental rat/mouse model of TLE NLRP1/caspase-1 signaling was demonstrated and the silencing of NLRP1 inflammasome was shown to be a potential treatment for TLE [127]. In a rat amygdala kindling-induced status epilepticus (SE) model, the levels of IL-1β, caspase-1 and NLRP3 were found to be increased, and neuroprotection was observed when NLRP3 inflammasome was knocked down in vivo by small interfering RNAs [128]. Hence it was proposed that NLRP3 may be a target for treatment in epilepsy. Besides, the neuronal loss induced by the pentylenetetrazol-induced epilepsy model was demonstrated to be significantly inhibited in NLRP3 knockout mice [129]. Likewise, levels of NLRP3 inflammasome were higher and positively correlated with endoplasmic reticulum stress-related markers in epileptogenic foci of TLE patients, and in the pilocarpine-induced SE mouse model the hippocampal the markers were decreased when the NLRP3 inflammasome was blocked with MCC950 which is a potent and specific small-molecule inhibitor of the NLRP3 pathway [130].

Finally, there is an interest to study migraine in the context of coronavirus disease 2019 (COVID-19) that is caused by acute respiratory syndrome coronavirus 2 (SARS-CoV-2) and abruptly altered lives worldwide in 2020 [131]. Although various risk factors for COVID-19 (e.g older age, male gender, and respiratory disease) are less frequent in migraineurs, it is notable that COVID-19 and migraine seem to share various comorbid diseases, including hypertension, coronary artery disease, and cardiovascular diseases [131, 132]. From an inflammasome perspective, there is growing evidence suggesting that common risk factors, such as hypertension, atherosclerosis, diabetes, or infections, induce the innate inflammasome complex that is also related to neurological disorders [132, 133]. Thus, it is tempting to speculate that the inflammasomes triggered by either intrinsic events or external SARS-CoV-2 could present a functional link between migraine and COVID-19. Most relevant to this review, the viroporins of SARS-CoV, such as protein E, have been shown to activate the NLRP3 inflammasome [134, 135] and are associated with COVID-19 severity in patients [136]. Viroporins are small virally-encoded ion channels that oligomerize on the membrane of host cells, leading to the formation of hydrophilic pores (Fig. 2). Although the viroporins that activate the inflammasome in SARS-CoV-2 could be different from SARS-CoV, studies investigating them, especially in relation to NLRP3 inflammasome activation, should be performed [135]. The main cause of inflammation in COVID-19 is the so-called “cytokine storm”, underlining the importance of the NLRP3 inflammasome. In addition, elevated levels of extracellular ATP induced by SARS-CoV-2 infection may trigger hyperactivation of P2X7 receptors leading to NLRP3 inflammasome stimulation as a key mediator of neuroinvasion and consequent neuroinflammatory processes [137]. Of note, headache is reported to be the presenting symptom in 6–10% of symptomatic COVID-19 patients [131, 138]. Besides, clinical data regarding shared vascular and inflammatory comorbid diseases revealed that migraine patients may be more vulnerable to COVID-19 [131]. In conclusion, it may well be that NLRP3 inflammasome activation may turn out be the link between COVID-19 and migraine [138], hence making the investigation of the possible link between inflammation and migraine even more relevant.

## Conclusion

Neuroinflammation is an important mechanism in various neurological disorders, and also in migraine and its comorbid diseases. In fact, evidence is accumulating that parenchymal neuroinflammation may be a relevant pathway shared between migraine, epilepsy, stroke, and COVID-19 infection. The discovery of the neuroinflammation’s critical role in migraine give new insights into the cause of the disease. The importance of the expression profile of inflammasome complexes in the CNS, and more importantly which type of inflammasome complex assembles on which occasion, remains to be determined. Also the roles of NLRP1, NLRP2 and AIM2 inflammasome complexes needs further study in migraine and its comorbid diseases. Neuroinflammatory pathways, specifically those involving inflammasome proteins, seem good candidates as treatment targets, and perhaps even biomarkers, in migraine. The therapeutic potential of compounds targeting NLRP3 inflammasome signaling pathways in migraine, although promising, remains to be assessed.

## Data Availability

NA

## Abbreviations

AA: Arachidonic acid
AMPK: AMP-activated protein kinase
ASC: Apoptosis-associated speck-like protein containing a caspase recruitment domain
ATP: Adenosine tri-phosphate
BBB: Blood-brain barrier
CARD: Caspase recruitment domain
CGRP: Calcitonin gene-related peptide
COVID-19: Coronavirus disease 2019
CSD: Cortical spreading depolarization
DAMPs: Danger-associated molecular patterns
D-βHB: D-beta hydroxybutirate
FHM: Familial hemiplegic migraine
GWAS: Genome-wide association study
HMGB1: High mobility group box 1
IL1-β: Interleukin 1-beta
IL-1RN: Interleukin 1 receptor antagonist
K^+^: Potassium ion
MA: Migraine with aura
MELAS: Mitochondrial encephalomyopathy, lactic acidosis and stroke-like episodes
MO: Migraine without aura
mtDNA: Mitochondrial DNA
mTLE: Mesial temporal lobe epilepsy
NF-κb: Nuclear factor-kappa B
NLRP3: Nucleotide-binding domain (NOD)-like receptor family pyrin domain containing 3
PAMPs: Pathogen-associated molecular patterns
PRRs: Pattern recognition receptor
PYD: Pyrin domain
ROS: Reactive oxygen species
SARS-CoV-2: Acute respiratory syndrome coronavirus 2
SE: Status epilepticus
siRNA: Small interfering RNA
SP: Substance P
TG: Trigeminal ganglion
TLR: Toll like receptor
TNF-α: Tumor necrosis factor alpha
TXNIP: Thioredoxin-interacting protein
WT: Wild type
